# Axillary surgical approach in metastatic breast cancer patients: a systematic review and meta-analysis

**DOI:** 10.3332/ecancer.2020.1117

**Published:** 2020-10-06

**Authors:** Fabiana C A P Lisboa1a, Roberta B Silva, Keitty R C de Andrade, Lucimara P C Veras, Ana Claudia M G Figueiredo, Maurício G Pereira

**Affiliations:** 1 Faculty of Medicine, University of Brasilia, Brasilia, Distrito Federal 70910-900, Brazil; 2 Nutritionist, Faculty of Health Sciences, University of Brasilia, Brasilia, Distrito Federal 70910-900, Brazil; 3 Physiotherapist, Faculty of Medicine, University of Brasilia, Brasilia, Distrito Federal 70910-900, Brazil; 4 Foundation of Education and Research in Health Sciences, Brasilia, Distrito Federal 70710-907, Brazil; 5 Faculty of Health Sciences, University of Brasilia, Brasilia, Distrito Federal, Brazil; 6 Faculty of Medicine, Brasilia, Distrito Federal 70910-900, Brazil; a https://orcid.org/0000-0002-3441-993X

**Keywords:** breast neoplasms, neoplasm metastasis, surgery, meta-analysis, systematic review

## Abstract

A systematic review and meta-analysis were conducted to evaluate the benefit of an axillary surgical approach on overall survival and secondarily of breast surgery amongst patients with metastatic breast cancer which is considered to be an incurable disease. However, an axillary surgical approach showed no association with overall survival in patients with metastatic breast cancer. The true impact of locoregional therapies on long-term outcomes remains unknown, and randomised clinical trials are needed.

## Introduction

Metastatic breast cancer affects approximately 3.5%–10% of patients at primary diagnosis [1, 2]. Although it is potentially treatable, the median overall survival is only about 2–3 years, and only 25% of the patients will still be alive after 5 years [3]. A systemic approach to treatment is recommended, and locoregional surgery is only indicated in symptomatic cases [4]. However, a surgical approach is an independent factor to improve survival and promote better local control, improving the quality of life and resulting in a mortality reduction [5]. Its use has also been linked to more than 10% of metastatic patients who live at least 10 years and reduces the risk of death by 30%–37% [6, 7].

A surgical approach in metastatic breast cancer is not the standard protocol, but 35%–60% of patients usually receive surgical treatment of the primary tumour [1, 2, 8, 9], and this proportion is higher for those receiving surgery for palliative purposes only [9]. However, observational studies have shown an increase in overall survival associated with surgical treatment despite being controversial data [8]. Prospective studies are expected to answer the questions as to whether there is a benefit in the surgical approach of the breast and/or armpit.

Uncontrolled disease present in the axilla may function as a constant source of disease spread [10]. The reason why an axillary approach to surgery is beneficial for survival is not clearly known, and its indication is controversial [11]. However, the self-dissemination of primary tumour cells to distant sites and an immune suppressive microenvironment can be reduced by surgical resection of the primary tumour combined with a concomitant axillary surgical approach [12]. It is questioned whether axillary emptying would bring greater comorbidity to a patient with a poor prognosis as in this metastatic scenario or whether it could bring benefits in terms of survival and local control.

The purpose of the study is the systematic review and meta-analysis of the axillary surgery approach in metastatic breast cancer (MBC) and secondarily of the breast surgery approach in this same scenario. According to the authors’ knowledge, this is the first systematic review and meta-analysis on the subject.

## Methods

### Registration and protocol

This systematic review was recorded in the International Prospective Register of Systematic Reviews (PROSPERO) under the number CRD42017077752 and was conducted based on the checklist for meta-analyses of observational studies (MOOSE) [13] (Appendix S1**—**MOOSE Checklist for Meta-analyses of Observational Studies) and preferred reporting items for systematic reviews and meta-analyses (PRISMA) (Appendix S2—PRISMA checklist) [14]. The Peer Review of Electronic Search Strategies (PRESS) checklist was applied, which is an instrument used for the independent peer review of the search strategy [15].

### Eligibility criteria

We included studies reporting patients who underwent axillary and breast surgical approaches in the presence of metastatic breast cancer and reported performance status for treatment and overall survival outcomes. Studies evaluating inflammatory breast cancer, enrolling those with the only supraclavicular disease or specific groups such as pregnant women, and letters or commentaries were excluded along with studies that had unavailable full texts and those that did not report hazard ratios (HRs) for overall survival.

### Information sources and search strategy

Literature searches were performed up to June 2018. The following databases were used: Medline (by PubMed), Embase, Clinical Trials, Scopus, Web of Science, SciELO, Lilacs, Google Scholar and ProQuest. The authors were contacted if additional information was required. Reference lists of the included records were hand-searched for additional eligible articles.

The search strategy was developed using MeSH terms for PubMed and EMTREE terms for EMBASE, as well as a combination of keywords for the other databases. The search strategy below was used on PubMed and, afterwards, adapted for each database (Appendix S1—Search strategy for each database):

((‘breast neoplasms’ [Mesh] OR ‘breast cancer’ OR ‘metastatic breast cancer’) AND (‘stage IV’ OR ‘stage IV breast cancer’ OR ‘stage IV metastatic breast cancer’) AND (‘lymph node excision’ [Mesh] OR ‘surgical excision’ OR surgery OR ‘local treatment’)).

No restriction of date or status of publication, language or type of study was applied**.**

### Study selection process

The study selection first consisted of assessing titles and abstracts after the removal of duplicate records. Eligibility assessment was performed by full-text evaluation, and those articles that did not appear to meet the eligibility criteria were excluded. Data from the selected articles were extracted to a Microsoft Excel® spreadsheet (2016). All steps were performed independently by two authors (FCAPL and RBS), and disagreements were resolved by consensus or with the intervention of a third reviewer (KRCA).

### Quality assessment of studies

The Joanna Briggs Institute tools were used to evaluate the methodological quality of the studies. The checklist for cohort studies [16] evaluated 11 questions related to the similarity between groups, exposure and outcome measures, strategies to control for confounding factors, absence or presence of the outcome at the beginning of follow-up, follow-up time and statistics. The checklist for randomised controlled trials [17] analyses 13 questions about randomisation, group allocation, blinding, intervention, statistical analysis and appropriateness of the study design. The greater the number of ‘yes’ answers, the higher the probability of having a good methodological quality.

### Quality of the evidence

To evaluate the quality of the evidence across studies through the GRADE evaluation [18], five items were verified that were reduced by two points per item the quality of the evidence: risk of bias, inconsistency, indirect evidence, inaccuracy and publication bias. In parallel, three items increased the quality of evidence by up to two points: magnitude of the effect, dose-response gradient and possible confounding adjustment. According to the GRADE classification, the evidence was considered to be of high quality when it reached at least four points, moderate quality at three points, low quality at two points and very low quality at one point [19].

### Risk of bias

We assessed the risk of bias using Cochrane’s risk of bias tool as described in the Cochrane Handbook for Systematic Reviews of Interventions [20].

### Data analysis

The primary endpoint was overall survival in the axillary surgery approach in MBC. The second endpoint was overall survival in breast surgery in this same scenario.

The estimated proportion and its respective confidence interval (95% CI) were considered for analysis. A Chi-square test (*χ*^2^) was applied amongst studies, with a *p*-value < 0.05. A sensitivity analysis was performed. The heterogeneity amongst studies was measured using the I-squared statistic (*I*^2^) [21]. The values of 75% indicate high heterogeneity, values of 50% moderate heterogeneity and <25% low heterogeneity [22]. Random effect meta-analyses were performed using the Der Simonian–Laird method [23] considering HR measures with 95% confidence intervals (95% CI). STATA® version 15 was used in all statistical analyses.

## Results

### Study selection

**Figure 1.**
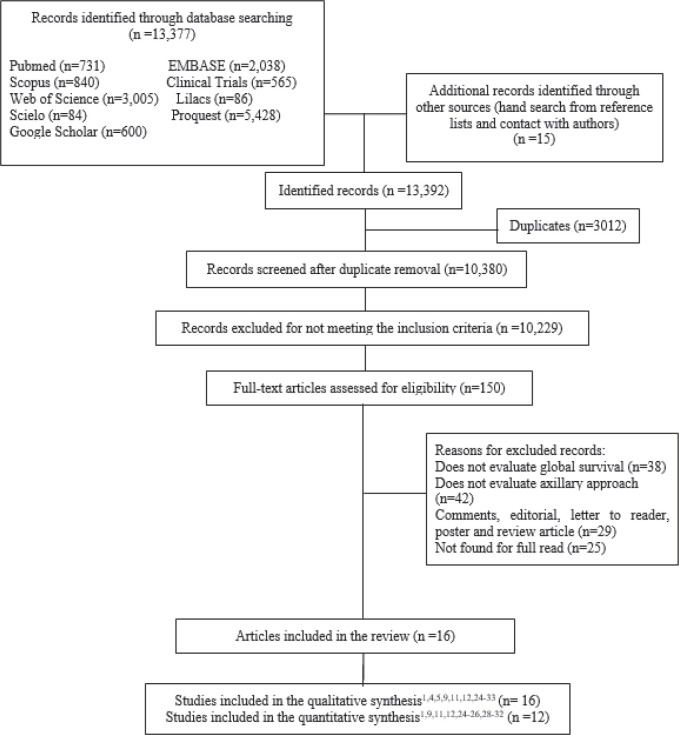
Flowchart of the study selection process.

A total of 13,377 studies were retrieved from database searches after the removal of duplicate records and title/abstracts screening (Figure 1). One hundred fifty full-text articles were assessed against the eligibility criteria. Finally, 16 studies were included in the qualitative analysis, with a total of 16,692 patients, and 12 studies were included in the quantitative analysis. The characteristics of the studies are shown in Table I (characteristics of the included studies).

### Study and participant characteristics.

Women were 97% of the sample [1, 4, 5, 9, 11, 12, 24, 27, 28, 30, 31, 32], and the median patient age at diagnosis was 57 years. The majority of participants were considered to be White (78%) [4, 12, 25, 27, 33]. A total of 9,504 patients underwent surgery, and amongst these, 55% underwent an axillary surgical approach. Only five studies included patients undergoing sentinel lymph node biopsy [1, 24, 25, 30, 32], and from these, one reported that 27% of the patients successfully underwent this procedure [24]. Mastectomy was the most prevalent surgery [1, 4, 9, 11, 25–33].

In the surgery group, bone metastasis was more common than visceral metastasis [5, 8, 9, 24–33], and single organ metastasis was more frequent than multiple metastases [1, 5, 8, 25, 27–33]. No prospective studies were found with the primary aim of comparing survival data due to axillary management [12]. There was a report on the reduction of the risk of death by up to 40% with the axillary surgical approach [34]. Complementary radiotherapy to surgery was performed on an average of 27% of patients [1, 4, 9, 24, 25, 26, 27, 28, 29, 31, 32], with only two studies citing survival results related to this procedure [10, 28]. Treatment with chemotherapy was performed by an average of 75% of patients, with only three studies discriminating against the scheme based on anthracycline [1, 5, 30], taxane [1, 27] and target therapy with trastuzumab [9, 30, 31].

It is noteworthy that the majority who underwent chemotherapy was in the surgery group, and the non-surgery group received hormone therapy more frequently. Systemic treatment regimens were diverse and carried out at different times, with more effective drugs used in the most current studies, which has an impact on survival. Radiotherapy was not performed and was mentioned uniformly in the research.

### Quality assessment

Hence, we chose to direct the information to respond to the purpose of the review, avoiding excesses and unclear initial data. The quality assessment for each study is shown in Table II (risk of bias assessed by the Joanna Briggs Institute’s critical appraisal checklist for cohort studies) and III (risk of bias assessed by the Joanna Briggs Institute’s critical appraisal checklist for randomised controlled trials). All studies seemed to have good methodological quality, with a mean of eight ‘yes’ answers.

### Quality of evidence

According to the evaluation of the Grade System [19], the classification of the evidence was of very low quality due to the high inconsistency across the studies, statistical inaccuracies and possibility of selection bias.

### Sensitivity analysis

The sensitivity analysis considered two studies’ outliers from the meta-analysis and were disregarded from the analysis which did not affect the results.

### Meta‑analyses for overall survival

**Figure 2.**
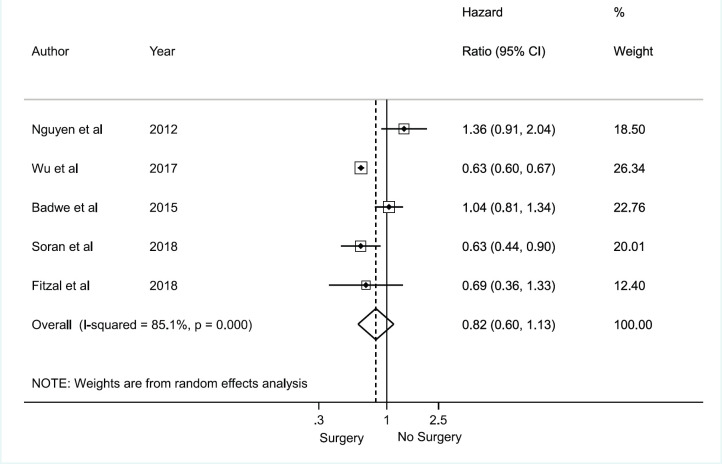
Meta-analysis for axillary approach and overall survival.

**Figure 3.**
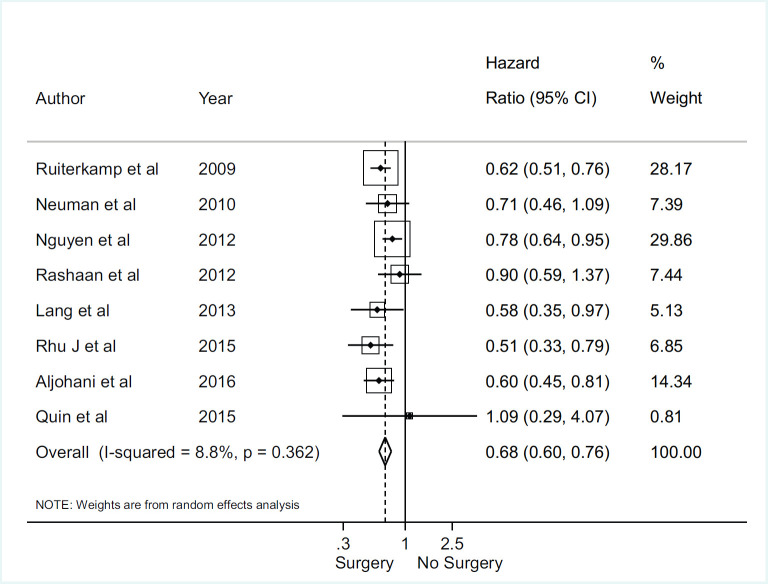
Meta-analysis for breast surgery and overall survival.

There was no association between axillary surgical resection and survival (HR 0.82; 95% CI, 0.60–1.13), and a high heterogeneity between studies was observed, with an *I*^2^ = 85.1% and *p* < 0.001 [1, 12, 30, 31, 32] (Figure 2). Regarding breast surgical resection [1, 9, 11, 24, 25, 26, 28, 29], there was a protective effect related to survival (HR 0.70; 95% CI, 0.60–0.82) with a moderate and significant heterogeneity (*I*^2^ = 40.5%, *p* = 0.098) (Figure 3).

### Risk of bias

**Figure 4.**
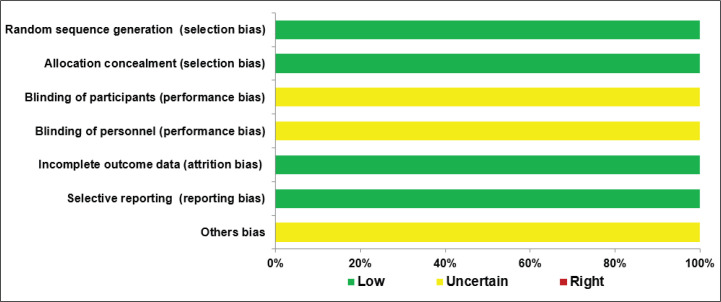
Cochrane’s risk of bias tool.

We used Cochrane’s tool for assessing the risk of bias, and the result revealed that the studies were at risk of low or uncertain bias as shown in Figure 4.

## Discussion

The available evidence indicated that the axillary surgical approach in metastatic breast cancer was not associated with an increase in overall survival. It was observed that the majority of participants with breast cancer underwent an axillary surgical technique. However, most of the physicians used this treatment preferably for women who presented with small tumours, and it is suspected that there may be great variability amongst women with metastatic breast cancer due to different stages of the disease and access to systemic treatment, which may impact the survival of the patients affected by the neoplasia.

Moreover, it was possible to determine, as a secondary result of this systematic review, an association between the surgical approach and an increase in overall survival. Mastectomy was the predominant surgical option, and the use of other techniques that would allow for breast reconstruction was not reported. There is a previously published systematic review that evaluated the relationship between breast surgery in women with metastasis and overall survival [35], but there are no reports on the influence of the axillary surgical approach on the outcome investigated.

Cohort studies, which were considered to be of good methodological quality, were included in this study [1, 12]; however, there are few longitudinal studies with robust data that evaluated the relationship between overall survival and local control of the disease after the use of axillary surgery. Omitting primary tumour surgery may reduce the overall survival of these patients [4, 29], but the relationship between the surgical technique and this outcome was not evaluated in any studies.

In addition, some authors have stated that women younger than 50 years, with smaller tumours (T1/T2), a lower volume of metastatic disease and those without HER-2/neu amplification and fewer comorbidities are more likely to be treated with aggressive multimodal therapy in the form of surgical excision of the primary tumour and local radiotherapy and systemic treatment [9, 11, 31, 32, 36]. However, it has been perceived that there is insufficient evidence to strengthen the use of these criteria as indications for axillary surgical treatment of metastatic breast cancer.

Two important randomised trials attempted to assess the effect on overall survival of locoregional treatment on the primary tumour in the breast and armpit compared to non-treatment in metastatic breast cancer [30, 31]. Both studies concluded that there is no evidence to suggest that locoregional treatment of the primary tumour affects overall survival in patients with metastatic breast cancer at the initial presentation. However, in the Turkish study, Soran 2018 showed benefit in terms of overall survival in subgroup analyses in selected patients with positive hormone receptor, negative HER-2, women under 55 years old and single bone metastasis [30].

The women included in the Indian study conducted by Badwe 2015 had more advanced diseases, more sites of metastasis and were mostly symptomatic, and only 8.5% of patients with HER2 tumour overexpression received anti-HER2 therapy [31]. These factors may reflect the inefficiency of the country’s health and screening programmes. However, it can interfere with the evaluation of the intervention [35]. In addition, the study excluded patients who did not respond to systemic treatment, i.e., patients with a worse prognosis. In the Turkish study, patients had fewer simultaneous metastasis sites, with better access to treatment with Trastuzumab for all patients with tumour overexpression of HER2. These aspects highlighted interfere in the overall survival result and may have contributed to a better result in subgroup analysis.

The systematic review of Tosello 2018 mapped randomised clinical trials, and it is not possible to do a meta-analysis because only two studies (Turkish and Indian) were included. The conclusion is that it is uncertain whether breast surgery improves overall survival since the quality of the evidence was assessed as very low [35]. It is known that, in the absence of randomised clinical trials, observational studies are considered as an alternative to initial evidence on the subject [37]. Still, it is delicate to carry out randomised clinical trials in vulnerable population groups, such as women with metastatic cancer. For this reason, there may be few randomised clinical trials on the topic.

Another prospective study with the same objective as the previous ones did not show any benefit in the overall survival for locoregional treatment in metastatic breast cancer, but the trial has stopped early due to insufficient recruitment [32]. The groups were well balanced as to the type of systemic treatment; however, in the surgery group, patients had more advanced tumours, both in the breast and in the armpit, which may have influenced the prognosis and, consequently, survival.

Locoregional treatment for metastatic cancer is hypothesised to improve survival based on retrospective analyses, but randomised studies provide conflicting data. Retrospective studies have peculiarities as women who were submitted to surgery belonged to younger groups, with smaller tumours, being a positive hormonal receptor, and presenting a lower volume of metastatic disease. The ASCO report (summary LB2A) of the randomised phase III trial E2108 suggests that early local therapy does not extend survival in patients with newly diagnosed metastatic breast cancer and has no benefit in terms of quality of life [38]. Locoregional treatment in this scenario can be considered when the systemic disease is well controlled, but the locoregional disease is progressing. Some data from this study are still pending and may help to clarify many issues in the future.

What differentiates the results of this review from other studies [30, 31, 35, 38] is the type of study employed, possibly being the first systematic review that used retrospective studies, due to the scarcity of randomised clinical trials on the topic [38].

From a methodological point of view, a randomised clinical trial is not an absolute truth to ensure that evidence is effective and applicable to the entire population. To elucidate these questions, systematic review is recommended, which was the objective of the present study.

Regarding the limitations, the possibility of selection bias of the participants in the original research is raised since aspects related to the use of systemic therapy can interfere with the evolution of the disease, in the decision-making about the surgical intervention and in the allocation of these women to the group treated with axillary surgery; that is, only women with a better prognosis may have been referred for surgery. In addition, the sample representation is another item that should be considered as a limitation of the original research. Although a broad search was conducted in several databases, there was only a limited amount of evidence available on the subject. Thus, it was not possible to apply other more robust analytical techniques such as evaluation of publication bias, Egger testing, subgroup analysis and meta-regressions.

A possible source of heterogeneity may have been the inclusion of methodologically different studies, diversity in the use of treatment protocols, insufficient registration of information in the original research, regional differences and the use of combined surgical techniques (breast and axillary approaches). Through the sensitivity analysis, it was possible to identify the major factors that influenced the high statistical heterogeneity.

From the perspective of reducing bias, a search was made of the grey literature, and contact was made with the authors of the published articles about missing data. Nevertheless, caution should be exercised in the interpretation of the findings since the data may present distorted results. The Grade assessment indicated a very poor quality of evidence due to the risk of bias, inaccuracy and inconsistency. Thus, the results of this review should be carefully evaluated before being considered as a recommendation.

Regarding strengths, the use of validated instruments for the sensitive evaluation of the analysis of search strategies, measurement of methodological quality and writing of systematic reviews, such as PRESS, Joanna Briggs, MOOSE and Prisma, was employed. Another positive aspect was the selection of cohorts, with a secure record of the intervention and outcome, minimising the possibility of information bias.

## Conclusion

As far as we know, this is the first systematic review on this topic, and we observed that there is no association between an axillary surgical approach and increased overall survival in patients with metastatic breast cancer. Thus, it is necessary to carry out additional longitudinal studies on this topic, with increased methodological robustness. The indications for the surgical approach in the metastatic context should be individualised considering the characteristics of the individual and the response to systemic treatment. The true impact of locoregional therapies on long-term outcomes remains unknown.

## Conflicts of interest

The authors state that they do not have any conflicts of interest.

## Funding statement

There are no sources of support for the reported work, including grants, equipment and medications, and no funding was received for this work from any organisation.

## Figures and Tables

**Table 1.** Characteristics of the included studies.

Author, publication year	Period of enrolment	Prospective study design	Country	Sample size (n)	Follow-up (months)	Axillary and breast surgery (n)	Overall survival axillary approach (surgery × not surgery)	Overall survival breast surgery (surgery × not surgery)	Mortality (surgery × not surgery)
Ruiterkamp *et al* [29]	1993–2004	No	Netherlands	728	156	BS 288/728 // ALND 190/288	ALND tended to have a better overall survival but only in the first year *p* = 0.35	HR = 0.62; 95% CI; 0.51–0.76 *p* < 0.001 31 versus 14 months 5-year survival rate 24.5% versus 13.1% *p* < 0.0001	Reduction of mortality risk ~ 40% HR = 0.66; 95% CI; 0.55–0.80
Mcguire *et al* [27]	1990–2007	No	USA	566	37	BS154/566// ALND 97/154	Nonsignificant trend toward ALND 46% versus 17% *p* = 0.26	OS rate 33% versus 20% *p* = 0.0012	-
Neuman *et al* [9]	2000–2004	No	USA	186	52	BS 69/186// ALND 33/69	-	HR = 0.71; 95% CI; 0.47–1.10; *p* = 0.10 40 versus 33 months 5-year survival rate 30%	69% died during follow-up
Nguyen *et al* [1]	1996–2005	Yes	Canada	733	22.8	BS 318/733// ALND + SLND 215/318	HR 1.36; 95% CI 0.91–2.04 5-year Kaplan–Meier OS 24.1 % versus 17.5% *p* = 0.31	HR=0.78; 95% CI; 0.64–0.94 *p* = 0.009 5-year Kaplan–Meier OS 21% versus 14% *p* < 0.001	-
Rashaan *et al* [11]	1989–2009	No	Netherlands	171	-	BS 59/171//ALND 43/59	-	HR = 0.90 95% CI 0.59–1.37 *p* = 0.05	Decreased risk of death with no comorbidity HR 0.4 95% CI 0.2–0.7 *p* = 0.002 and no medication use HR=0.5 95% CI; 0.2–0.8 *p* = 0.01
Sofi *et al* [33]	1999–2009	No	USA	37	-	BS 37 // ALND 37	-	8.83 versus (4.91 + 2.26) years *p* = 0.005	-
Lang *et al* [25]	1997–2002	No	USA	208	74.2	BS 74/208 // ALND or SLND 39/74	No significant trend toward improved survival to axillary procedure (SLND or ALND) *p* = 0.06	HR = 0.58, 95 % CI 0.35–0.98 *p* = 0.04	65% died during follow-up
Elkhouly [5]	2009–2011	No	Egypt	151	-	BS 61/151// ALND 60/61	-	39.10 versus 28.04 months <*p* = 0.001	89% × 98% *p* = 0.156 45% died during follow-up
Rhu *et al* [26]	1995–2011	No	Korea	262	29.6	BS 40/262// ALND 33/40	-	HR 0.51; 95% CI; 0.33–0.80) *p* < 0.01 Global mean survival rate 55 months (CI 41.81–68.25)	65% versus 69% *p* = 0.58
Quinn *et al* [24]	2006–2012	No	Ireland	109	24	BS 52/109 // ALND 45 and SLND 14	SLNB 20.2 and ALND 34.8 months *p* = 0.363	HR=1.094; 95% CI; 0.442–6.128 *p* = 0.013 Mean OS 29.5 months 35.8 versus 20.2 months *p* = 0.003	-
Badwe *et al* [31]	2001–2012	Yes	India	350	23	BS 165 ALND 165	HR 1.94 (0.81 – 1.34) *p* = 0,79	-	50.2% versus 49.8%
Aljohani *et al* [28]	2000–2012	No	Saudi Arabia	678	41	BS 412/678 // ALND 264/678	-	HR 0.60 95% CI 0.48–0.873 *p* = 0.0003 5-year survival rate 34% versus 14% // 41 versus 27 months *p* < 0.0029	52% died during follow-up
Wu *et al* [12]	1990–2010	No	China	11.645	-	BS – // ALND 7358 / 11645	OS HR = 0.630; 95% CI; 0.60–0.66; *p* < 0.001 BCSS HR = 0.633; 95% CI; 0.60–0.67; *p* < 0.001	-	-
Muzaffar *et al* [4]	1988–2011	No	USA	439	48	BS 222/439 // ALND 117/222	-	Median OS 29 versus 11 months Not surgery diminished survival HR = 1.81; 95% 1.42–2.31; *p* < 0.001	-
Soran *et al* [30]	2000–2012	Yes	Turkey	274	54.5	BS 138 SLND 23 (17%) ALND 128 (92.8%)	HR 0.63 (0.44 – 0.90) *p* = 0.008	-	55% versus 74% 34% lower in the surgery group
Fitzal *et al* [32]	2011–2015	Yes	Austria	90	37.5	BS 42 SLND 3 (7.1%) EA 39 (93%)	HR 0.691 (0.358 – 1.333) *p* = 0.267	-	-

BS – breast surgery,

ALND – axillary lymph node dissection,

SLND – sentinel lymph node biopsy,

HR – hazard ratio,

95% CI – 95% confidence interval,

OS – overall survival,

BCSS – breast cancer-specific survival.

**Table 2.** Risk of bias assessed by Joanna Briggs Institute’s critical appraisal checklist for cohort studies.

Questions	Ruiterkamp *et al* [29]	McGuire *et al* [27]	Neuman *et al* [9]	Nguyen *et al* [1]	Rashaan *et al* [11]	Sofi *et al* [33]	Lang *et al* [25]	Elkhouly [5]	Rhu J *et al* [26]	Quinn *et al* [24]	Aljohani *et al* [28]	Wu *et al* [12]	Muzaffar *et al* [4]
1. Were the two groups similar and recruited from the same population?	U	U	Y	Y	N	N	Y	N	Y	Y	N	Y	N
2. Were the exposures measured similarly to assign people to both exposed and unexposed groups?	N	Y	Y	U	N	N	U	N	U	Y	N	N	U
3. Was the exposure measured in a valid and reliable way?	Y	Y	Y	Y	N	Y	Y	U	Y	Y	Y	Y	Y
4. Were confounding factors identified?	Y	Y	Y	Y	Y	Y	Y	Y	Y	Y	Y	Y	Y
5. Were strategies to address confounding factors stated?	Y	N	Y	Y	Y	Y	Y	Y	Y	Y	U	Y	Y
6. Were the groups/participants free of the outcome at the start of the study (or at the moment of exposure)?	Y	Y	Y	Y	Y	Y	Y	Y	Y	Y	Y	Y	Y
7. Were the outcomes measured in a valid and reliable way?	Y	Y	Y	Y	Y	Y	Y	U	Y	U	Y	Y	U
8. Was the follow-up time reported and sufficient to be long enough for outcomes to occur?	Y	Y	Y	N	N	Y	Y	Y	Y	Y	Y	Y	Y
9. Was follow-up complete, and if not, were the reasons to loss to follow-up described and explored?	Y	N	N	N	N	Y	N	N	N	N	N	N	N
10. Were strategies to address incomplete follow-up utilised?	Y	N	Y	Y	Y	Y	Y	Y	Y	N	Y	Y	Y
11. Was appropriate statistical analysis used?	Y	N	Y	Y	Y	Y	Y	Y	Y	Y	Y	Y	Y
Total number of ‘y’ answers	9	6	10	8	6	9	9	10	6	8	9	7	7

Note: Y = yes; N = No; U = unclear.

**Table 3.** Risk of bias assessed by the Joanna Briggs Institute’s critical appraisal checklist for randomised controlled trials.

Questions	Badwe *et al* [31]	Soran *et al* [30]	Fitzal el al. [32]
1. Was true randomisation used for the assignment of participants to treatment groups?	Y	Y	Y
2. Was allocation to treatment groups concealed?	Y	Y	y
3. Were treatment groups similar at the baseline?	Y	Y	N
4. Were participants blind to treatment assignment?	N	N	N
5. Were those delivering treatment blind to treatment assignment?	N	N	N
6. Were outcomes assessors blind to treatment assignment?	N	N	N
7. Were treatment groups treated identically other than the intervention of interest?	Y	Y	Y
8. Was follow-up complete, and if not, were differences between groups in terms of their follow-up adequately described and analysed?	Y	Y	N
9. Were participants analysed in the group to which they were randomised?	Y	Y	Y
10. Were outcomes measured in the same way for treatment groups?	Y	Y	Y
11. Were outcomes measured in a reliable way?	Y	Y	Y
12. Was appropriate statistical analysis used?	Y	Y	Y
13. Was the trial design appropriate and any deviations from the standard RCT design (individual randomisation and parallel groups) accounted for in the conduct and analysis of the trial?	Y	Y	Y
Total number of ‘y’ answers	10	10	8

Note: Y = yes; N = No; U = unclear

## Appendix

**Appendix S1.** MOOSE checklist for meta-analyses of observational studies.

Item no	Recommendation		Reported on page no
Reporting of background should include			
1	Problem definition		4
2	Hypothesis statement		4
3	Description of study outcome(s)		4
4	Type of exposure or intervention used		4
5	Type of study designs used		4
6	Study population		4
Reporting of search strategy should include			
7		Qualifications of searchers (e.g., librarians and investigators)	1
8		Search strategy, including time period included in the synthesis and keywords	3;4;5
9		Effort to include all available studies, including contact with authors	4;5
10		Databases and registries searched	5
11		Search software used, name and version, including special features used (e.g., explosion)	7
12		Use of hand searching (e.g., reference lists of obtained articles)	5
13		List of citations located and those excluded, including justification	4
14		Method of addressing articles published in languages other than English	5
15		Method of handling abstracts and unpublished studies	5
16		Description of any contact with authors	4;5
Reporting of methods should include			
17	Description of relevance or appropriateness of studies assembled for assessing the hypothesis to be tested		6
18	Rationale for the selection and coding of data (e.g., sound clinical principles or convenience)		3;4
19	Documentation of how data were classified and coded (e.g., multiple raters, blinding and inter-rater reliability)		4
20	Assessment of confounding (e.g., comparability of cases and controls in studies where appropriate)		-
21	Assessment of study quality, including blinding of quality assessors, stratification or regression on possible predictors of study results		6
22	Assessment of heterogeneity		6,7,8
23	Description of statistical methods (e.g., complete description of fixed or random-effects models, justification of whether the chosen models account for predictors of study results, dose-response models or cumulative meta-analysis) in sufficient detail to be replicated		7
24	Provision of appropriate tables and graphics		16-27
Reporting of results should include			
25	Graphic summarising individual study estimates and overall estimate		25–26
26	Table giving descriptive information for each study included		17
27	Results of sensitivity testing (e.g., subgroup analysis)		8
28	Indication of statistical uncertainty of findings		11

**Appendix S2.** PRISMA checklist.

Section/topic	#	Checklist item	Reported on page #
**TITLE**			
Title	1	Identify the report as a systematic review, meta-analysis or both.	1
**ABSTRACT**			
Structured summary	2	Provide a structured summary including as applicable: background; objectives; data sources; study eligibility criteria, participants and interventions; study appraisal and synthesis methods; results; limitations; conclusions and implications of key findings and systematic review registration number.	2
**INTRODUCTION**			
Rationale	3	Describe the rationale for the review in the context of what is already known.	4
Objectives	4	Provide an explicit statement of questions being addressed with reference to participants, interventions, comparisons, outcomes and study design (PICOS).	4
**METHODS**			
Protocol and registration	5	Indicate if a review protocol exists, if and where it can be accessed (e.g., Web address), and if available, provide registration information including registration number.	4
Eligibility criteria	6	Specify study characteristics (e.g., PICOS and length of follow-up) and report characteristics (e.g., years considered, language and publication status) used as criteria for eligibility, giving rationale.	4
Information sources	7	Describe all information sources (e.g., databases with dates of coverage and contact with study authors to identify additional studies) in the search and date last searched.	5
Search	8	Present full electronic search strategy for at least one database, including any limits used, such that it could be repeated.	5
Study selection	9	State the process for selecting studies (i.e., screening, eligibility, included in systematic review, and, if applicable, included in the meta-analysis).	5
Data collection process	10	Describe the method of data extraction from reports (e.g., piloted forms, independently, in duplicate) and any processes for obtaining and confirming data from investigators.	5
Data items	11	List and define all variables, for which data were sought (e.g., PICOS and funding sources) and any assumptions and simplifications made.	1;4
Risk of bias in individual studies	12	Describe methods used for assessing the risk of bias of individual studies (including specification of whether this was done at the study or outcome level), and how this information is to be used in any data synthesis.	6
Summary measures	13	State the principal summary measures (e.g., risk ratio and difference in means).	7
Synthesis of results	14	Describe the methods of handling data and combining results of studies, if done, including measures of consistency (e.g., I^2^) for each meta-analysis.	7
Risk of bias across studies	15	Specify any assessment of the risk of bias that may affect the cumulative evidence (e.g., publication bias and selective reporting within studies).	8
Additional analyses	16	Describe the methods of additional analyses (e.g., sensitivity or subgroup analyses and meta-regression), if done, indicating which were pre-specified.	7
**RESULTS**			
Study selection	17	Give the number of studies screened, assessed for eligibility and included in the review, with reasons for exclusions at each stage, ideally with a flow diagram.	7
Study characteristics	18	For each study, the present characteristics for which data were extracted (e.g., study size, PICOS and follow-up period) and provide the citations.	7
Risk of bias within studies	19	Present data on the risk of bias of each study and, if available, any outcome level assessment (see item 12).	8
Results of individual studies	20	For all outcomes considered (benefits or harms), present, for each study: (a) simple summary data for each intervention group (b) effect estimates and confidence intervals, ideally with a forest plot.	8
Synthesis of results	21	Present results of each meta-analysis done, including confidence intervals and measures of consistency.	8
Risk of bias across studies	22	Present results of any assessment of the risk of bias across studies (see Item 15).	8
Additional analysis	23	Give results of additional analyses, if done (e.g., sensitivity or subgroup analyses and meta-regression [see Item 16]).	8
**DISCUSSION**			
Summary of evidence	24	Summarise the main findings including the strength of evidence for each main outcome; consider their relevance to key groups (e.g., healthcare providers, users and policymakers).	9
Limitations	25	Discuss limitations at the study and outcome level (e.g., risk of bias) and at the review level (e.g., incomplete retrieval of identified research and reporting bias).	10
Conclusions	26	Provide a general interpretation of the results in the context of other evidence and implications for future research.	11
**FUNDING**			
Funding	27	Describe sources of funding for the systematic review and other support (e.g., supply of data); the role of funders for the systematic review.	1

*From:* Moher D, Liberati A, Tetzlaff J, Altman DG, The PRISMA Group (2009). Preferred Reporting Items for Systematic Reviews and Meta-Analyses: The PRISMA Statement. PLoS Med 6(6): e1000097. doi:10.1371/journal.pmed1000097

**Appendix S3.** Search strategy for each database.

Database	Search Strategy
PubMed	((‘breast neoplasms’ [Mesh] OR ‘breast cancer’ OR ‘metastatic breast cancer’) AND ‘stage IV’) OR ‘stage IV breast cancer’ OR (‘stage IV metastatic breast cancer’) AND (‘lymph node excision’ [Mesh] OR ‘surgical excision’ OR surgery OR ‘local treatment’)
Embase	(‘breast’/exp OR breast AND (‘tumour’/exp OR tumour) AND (‘breast cancer’ OR ‘metastatic breast cancer’) AND ‘stage IV’ OR ‘stage IV breast cancer’ OR ‘stage IV metastatic breast cancer’) AND (‘lymph node dissection’/exp OR ‘surgical excision’ OR surgery OR ‘local treatment’)
LILACS	(tw: (breast neoplasms OR breast cancer OR metastatic breast cancer OR stage IV metastatic breast cancer)) AND (stw: (lymph node excision OR surgical excision OR surgery OR local treatment))
SciELO	(breast cancer OR metastatic breast cancer) AND (surgery OR surgical excision)
Web of Science	(((‘breast neoplasms’ OR ‘breast cancer’ OR ‘metastatic breast cancer’) AND ‘stage IV’) OR ‘stage IV breast cancer’ OR (‘stage IV metastatic breast cancer’) AND (‘lymph node excision’ OR ‘surgical excision’ OR surgery OR ‘local treatment’))
Scopus Google Scholar Proquest	TITLE-ABS-KEY ((((‘breast neoplasms’ OR ’breast cancer’ OR ’metastatic breast cancer’) AND ’stage IV’) OR ’stage IV breast cancer’ OR (‘stage IV metastatic breast cancer’) AND (‘lymph node excision’ OR ’surgical excision’ OR surgery OR ’local treatment’))) (‘breast neoplasms’ OR ‘breast cancer’ OR ‘metastatic breast cancer’ OR ‘stage IV breast cancer’ OR ‘stage IV metastatic breast cancer’) AND (‘lymph node excision’ OR ‘surgical excision’ OR surgery OR ‘local treatment’) ((‘breast neoplasms’ OR ‘breast cancer’ OR ‘metastatic breast cancer’) AND ‘stage IV’) OR ‘stage IV breast cancer’ OR (‘stage IV metastatic breast cancer’) AND (‘lymph node excision’ OR ‘surgical excision’ OR surgery OR ‘local treatment’)
Clinical Trials	(‘breast neoplasms’ OR ‘breast cancer’ OR ‘metastatic breast cancer’ OR ‘stage IV breast cancer’ OR ‘stage IV metastatic breast cancer’) AND (‘lymph node excision’ OR ‘surgical excision’ OR surgery OR ‘local treatment’)
